# Hepatocyte growth factor and invasion-stimulatory activity are induced in pleural fluid by surgery in lung cancer patients

**DOI:** 10.1038/sj.bjc.6690754

**Published:** 1999-10

**Authors:** A Uchiyama, T Morisaki, K Beppu, M Kojima, Y Matsunari, A Nakatsuka, K Mizumoto, K Matsumoto, T Nakamura, M Tanaka

**Affiliations:** Department of Surgery I, Kyushu University Faculty of Medicine, Fukuoka , 812-8582, Japan; Division of Biochemistry, Department of Oncology, Biomedical Center, Osaka University Faculty of Medicine, Osaka, Japan

**Keywords:** hepatocyte growth factor, lung surgery, cancer invasion

## Abstract

Hepatocyte growth factor (HGF) is a stromal cell-derived cytokine that can stimulate matrix invasion by carcinoma cells. We analysed the concentrations of HGF and invasion-stimulatory activity in pleural fluid after lung surgery. The concentration of HGF in pleural fluids was measured by enzyme-linked immunosorbent assay in seven patients who underwent pulmonary resection for primary or metastatic lung cancer. The effect of the pleural fluid on cancer cell invasion across reconstituted basement membrane (Matrigel) was assessed with a Boyden chamber assay using a lung adenocarcinoma cell line, A549. HGF levels in the pleural fluid after lung surgery ranged from 6.0 to 23.0 ng ml^−1^ (average: 10.2 ± 4.3 ng ml^−1^). The matrix invasion of lung carcinoma cells in the presence of the pleural fluid was significantly higher than that in the presence of culture medium alone or sera from normal subjects (*P* < 0.01). The invasion-stimulatory activity of the pleural fluid was strongly inhibited by HGF-neutralizing antibody. Positive correlation was found between the HGF level and invasion-stimulatory activity in the pleural fluids and normal sera (*P* = 0.0073). This is the first report demonstrating that the lung surgery induces a considerable amount of HGF, which is closely correlated with the invasion-stimulatory activity of the pleural fluid. © 1999 Cancer Research Campaign


British Journal of Cancer (1999) **81**(4), 721–726
© 1999 Cancer Research Campaign
Article no. bjoc.1999.0754

### Hepatocyte growth factor and invasion-stimulatory activity are induced in pleural fluid by surgery in lung cancer patients

**A Uchiyama,[1] T Morisaki,[1] K Beppu,[1] M Kojima,[1] Y Matsunari,[1] A Nakatsuka,[1] K Mizumoto,[1] K Matsumoto,[2] T Nakamura[2] and M Tanaka[1]**

[1]Department of Surgery I, Kyushu University Faculty of Medicine, Fukuoka 812-8582, Japan; [2]Division of Biochemistry, Department of Oncology, Biomedical Center, Osaka University Faculty of Medicine, Osaka, Japan

**Summary** Hepatocyte growth factor (HGF) is a stromal cell-derived cytokine that can stimulate matrix invasion by carcinoma cells. We analysed the concentrations of HGF and invasion-stimulatory activity in pleural fluid after lung surgery. The concentration of HGF in pleural fluids was measured by enzyme-linked immunosorbent assay in seven patients who underwent pulmonary resection for primary or metastatic lung cancer. The effect of the pleural fluid on cancer cell invasion across reconstituted basement membrane (Matrigel) was assessed with a Boyden chamber assay using a lung adenocarcinoma cell line, A549. HGF levels in the pleural fluid after lung surgery ranged from 6.0 to 23.0 ng $ml^{-1}$ (average: 10.2 ± 4.3 ng $ml^{-1}$). The matrix invasion of lung carcinoma cells in the presence of the pleural fluid was significantly higher than that in the presence of culture medium alone or sera from normal subjects ($P < 0.01$). The invasion-stimulatory activity of the pleural fluid was strongly inhibited by HGF-neutralizing antibody. Positive correlation was found between the HGF level and invasion-stimulatory activity in the pleural fluids and normal sera ($P = 0.0073$). This is the first report demonstrating that the lung surgery induces a considerable amount of HGF, which is closely correlated with the invasion-stimulatory activity of the pleural fluid. © 1999 Cancer Research Campaign

**Keywords**: hepatocyte growth factor; lung surgery; cancer invasion

Acceleration of cancer progression after surgery is frequently encountered in clinical settings and is reproducible in animal experimental models (Kodama et al, 1992; Bogden et al, 1997). Surgical procedures cause tissue injuries resulting in release of various cytokines, or growth factors, in wound fluid. If residual cancer cells are present near the resection margin or in the fluid space, they are directly influenced by mitogenic or motogenic humoural factors in the wound environment.

Hepatocyte growth factor (HGF) was first identified (Nakamura et al, 1984) and cloned (Miyazawa et al, 1989; Nakamura et al, 1989) as a potent mitogen for cultured hepatocytes. Recent studies have shown that the HGF has a number of biological activities; for example, mitogenic, motogenic, and morphogenic (Zarnegar and Michalopoulos 1995; Matsumoto and Nakamura 1997). HGF is produced by stromal or mesenchymal cells (Noji et al, 1990; Matsumoto et al, 1992; Sonnenberg et al, 1993), and targets a wide variety of cells (Weidner et al, 1990; Zarnegar and Michalopoulos, 1995). It enhances the invasion and metastasis of cancer cells that express an HGF receptor, a c-met proto-oncogene product (Giordano et al, 1993; Jeffers et al, 1996). HGF is a stromal-derived polypeptide that affects invasion of carcinoma cells via tumour–stromal interactions (Matsumoto et al, 1996; Nakamura et al, 1997). We have recently shown that HGF stimulates matrix metalloproteinase (MMP)-1 and MMP-2 production in human colon carcinoma cells (Uchiyama et al, 1996). On the other hand, HGF plays a role in enhancing regeneration of organs such as liver, kidney and lung. The expression of HGF is rapidly induced following injuries in these organs (Zarnegar and Michalopoulos, 1995; Matsumoto and Nakamura, 1997). These findings suggest that HGF may be secreted in the wound microenvironment after some surgical procedures, and that the HGF may play a role in progression of residual cancer cells.

Kimura et al (1996) have reported secretion of HGF in the wound fluid in hepatic resection surgery. However, little is known about HGF secretion in the pleural fluid after lung surgery. This is an important point to consider, because the lung is thought to be one of the key organs that produces HGF (Yanagita et al, 1992), and an association of HGF with progression of non-small-cell lung cancer has recently been reported (Siegfried et al, 1997). In the study presented here, we measured HGF concentration in pleural fluid after lung surgery, and examined whether HGF in the pleural fluid can stimulate matrix invasion by lung carcinoma cells in vitro.

*Received 24 November 1998*
*Revised 5 May 1999*
*Accepted 5 May 1999*

*Correspondence to:* A Uchiyama

### MATERIALS AND METHODS

### Collection of samples

Pleural fluid was obtained from thoracic drain tubes from seven cases of pulmonary resection surgery for primary or metastatic lung cancer at the Department of Surgery I, Kyushu University Faculty of Medicine. The present study was carried out with the approval of the ethical committee organized by senior staffs in the Department of Surgery I. Informed consent for collecting serum

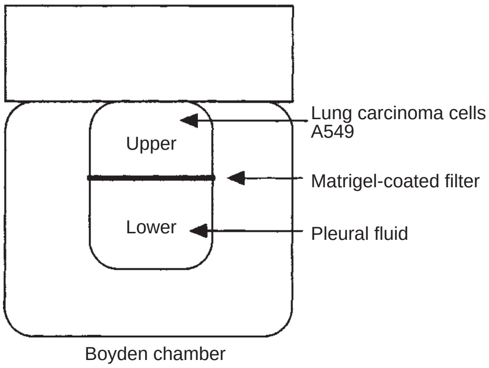

**Figure 1** Schema of the in vitro invasion assay system. The assay was performed as described in Materials and Methods and pleural fluid samples for research was obtained from each patient before surgery. The serum samples were collected simultaneously from peripheral blood. The supernantants of the samples were collected by centrifugation at 2200 rpm for 20 min and were stored at –80°C until assay.

### Enzyme-linked immunosorbent assay

HGF concentrations in the samples were measured with the aid of an HGF enzyme-linked immunosorbent assay (ELISA) kit (Institute of Immunology, Tokyo, Japan) following the manufacturer's instructions. Fivefold diluted pleural fluids and twofold diluted sera were used for the measurement of HGF concentration. The detection limit of this assay is 0.10 ng $ml^{-1}$.

### Cell line

A human lung adenocarcinoma cell line A549 was purchased from the Japanese Cancer Research Resources Bank. The cells were maintained in RPMI-1640 medium (Nissui, Tokyo, Japan) supplemented with 10% fetal bovine serum (Gibco-BRL, NY, USA) and antibiotics.

### Human recombinant HGF and HGF-neutralizing antibody

Human recombinant HGF was purified from the culture medium of Chinese hamster ovary cells transfected with an expression plasmid containing human HGF cDNA (Nakamura et al, 1989). Neutralizing polyclonal antibody against HGF was prepared from rabbits immunized with the human recombinant HGF (Matsumoto et al, 1996; Nakamura et al, 1997).

### In vitro invasion assay

The invasive response of carcinoma cells to the pleural fluid was assessed with the aid of blind-well chemotaxis Boyden chambers as described by Sieuwerts et al (1997) with some modifications. Figure 1 shows the schema of this assay system. Both upper and lower sides of polycarbonate filters (8-μm pore size, 13-mm in diameter; Nucleopore, CA, USA) were coated with Matrigel (Collaborative Research Inc., Bedford, MA, USA). The lower side of the filter was first coated with Matrigel to promote attachment of the cells. Thirty-microlitre drops of Matrigel (2 mg $ml^{-1}$ in serum-free medium) were placed in plastic dishes. Filters were placed on top of the drop of Matrigel. After 1 h incubation at 37°C, the upper side of the filter was coated with 35 μl of Matrigel and placed in an incubator for 1 h to allow the Matrigel to form an even gel layer on the filter. The lower compartment of the Boyden chambers was filled with either 225 μl of medium alone, sera obtained from normal subjects, or diluted pleural fluid samples. The coated filters were placed in the Boyden chambers. A549 lung carcinoma cells ($4 \times 10^4$ cells per well) suspended in the growth medium were added to the upper compartment and incubated for 24 h at 37°C in atmosphere of 5% carbon dioxide in air. At the end of the incubation period the non-invasive cells, which remained on the top of the filter, were removed with a cotton swab and the invasive cells, which were attached to the underside of the filter, were stained with Diff-Quick staining solutions (Baxter, McGaw Park, IL, USA). The cells attached to the lower surface were counted in a total of five microscopic fields (×200), and each assay was done in triplicate. The number of the invading cells per five fields was used as a measure of invasion-stimulatory activity of the samples.

**Table 1** Clinical characteristics of patients from whom pleural fluids were collected after pulmonary resection surgery

| Case | Age | Sex | Diagnosis | Stage | Operation | Postoperative complication | HGF level (ng/ml) in pleural fluid after surgery (POD[a]) |
|---|---|---|---|---|---|---|---|
| 1. | 70 | F | Lung cancer | IIB | Right upper lobectomy | None | 15.0 (Day 1), 9.2 (Day 2), 6.2 (Day 3) |
| 2. | 47 | M | Lung cancer | IIB | Left upper lobectomy | None | 12.4 (Day 1) |
| 3. | 70 | M | Lung cancer | I | Left lower lobectomy | None | 9.5 (Day 1), 9.6 (Day 3), 6.0 (Day 4) |
| 4. | 69 | M | Lung cancer | I | Right upper lobectomy | None | 5.2 (Day 1), 4.2 (Day 2), 11.2 (Day 3), 23.0 (Day 5), 11.5 (Day 6), 7.0 (Day 7) |
| 5. | 60 | F | Colon cancer Lung metastasis | IV | Right lower lobectomy | None | 18.0 (Day 2) |
| 6. | 70 | M | Colon cancer | IV | 1) Right partial resection | None | 15.5 (Day 2), 7.5 (Day 4) |
| | | | Lung metastasis | | 2) Left partal resection | None | 10.0 (Day 1) |
| | | | | | 3) Left lower lobectomy | Air leak | 11.0 (Day 3), 8.5 (Day 6) |
| 7. | 67 | M | Rectal cancer | IV | 1) Right partial resection | None | 8.0 (Day 1) |
| | | | Lung metastasis | | 2) Left partial resection | None | 7.2 (Day 1), 9.2 (Day 2), 10.4 (Day 3) |

[a]POD (post-operative day) at sample collection.

 © 1999 Cancer Research Campaign

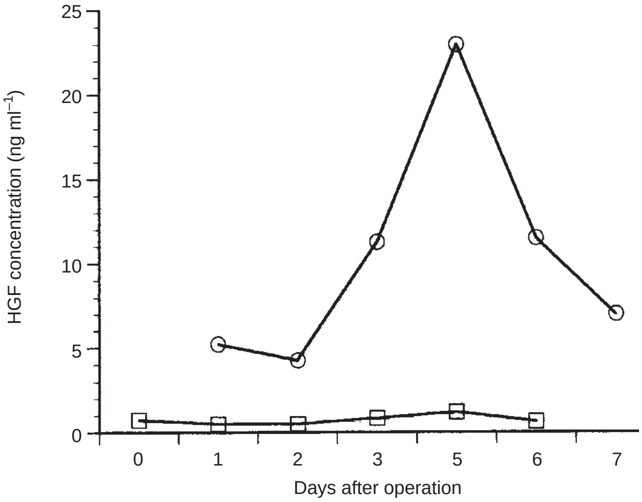

**Figure 2** Time course of HGF concentration in serum (open square) and pleural fluid (open circle) after operation in case 4. The HGF levels in the pleural fluids reached a maximum value on POD 5 after surgery and decreased slowly afterwards

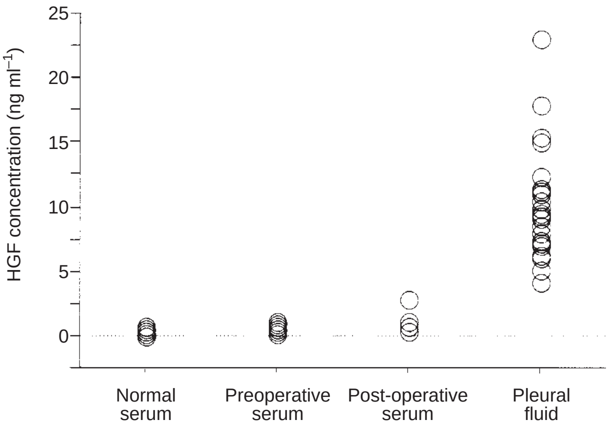

**Figure 3** HGF concentrations in the sera of normal subjects and patients before and after operation, and in the pleural fluid after lung surgery. *Significant difference by Kruskal-Wallis ANOVA test ($P$ < 0.0001)

### Statistical analyses

The Kruskal-Wallis ANOVA test was used for assessing the significance of differences in HGF concentration between the pleural fluid and the serum samples. Student's $t$-test was used for analysis of unpaired samples. Correlation was assessed using the Spearman's rank correlation test. Differences at the probability value less than 0.05 were considered significant.

### RESULTS

Pleural fluid samples were obtained from ten pulmonary resections from seven patients with primary or metastatic lung cancer. Table 1 shows patients' characteristics. None of them had pleural effusion before surgery. Patients 6 and 7 were operated on more than once. No cases had post-operative complications related to infection. In patient 4, both serum and pleural HGF levels were measured for 7 days. The HGF concentration in each pleural fluid sample is also shown in Table 1.

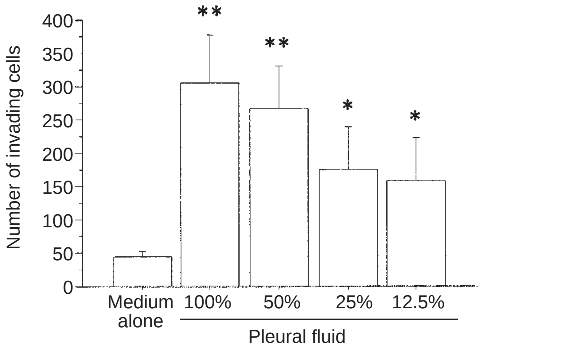

**Figure 4** Invasion of lung carcinoma cell line A549 across reconstituted basement membrane (Matrigel) in the presence of the serially-diluted pleural fluid after lung surgery. The pleural fluid sample was obtained on POD 6 after the third operation in case 6. The results of triplicate experiments are shown. The value was compared with that in the presence of medium alone. **$P$ <0.01, *$P$ <0.05

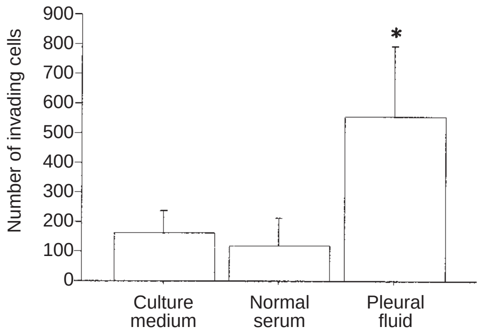

**Figure 5** The matrix invasion by lung carcinoma cells in the presence of the culture medium alone ($n$ = 6), twofold-diluted sera from normal subjects ($n$ = 4), and twofold-diluted pleural fluid after surgery ($n$ = 7). The pleural fluid samples used in this experiment were as follows: POD 1 in patient 1, POD 1 in patient 2, POD 1 and 4 in patient 3, POD 3 in patient 4, POD 3 after the third operation in patient 6, and POD 1 after the second operation in patient 7. The invasion was significantly higher in the presence of pleural fluid compared with that in the presence of culture medium and normal sera by Kruskal–Wallis ANOVA test. *$P$ < 0.01

In the patients 1, 3, 4 and 7 (Table 1), the HGF concentrations in the pleural fluids were measured on 3–6 postoperative day points. Figure 2 shows a time course of pleural and serum HGF levels after lung surgery in patient 4. The HGF levels in the pleural fluids reached a maximum value on post-operative day (POD) 5 after surgery and decreased slowly afterwards. The pleural HGF levels were higher than the serum HGF levels during the 7 days after operation. The post-operative day point with maximum HGF level varied from POD 1 to POD 5. The similar time course of pleural HGF level after lung surgery was observed in our preliminary experiments (data not shown).

The HGF concentrations in 23 samples of the pleural fluid ranged from 6.0 to 23.0 ng $ml^{-1}$ with an average of 10.2 ± 4.3 (s.d.) ng $ml^{-1}$ (Figure 3). Because we had no control over HGF level in the pleural fluid, the data were compared with the serum HGF levels in normal subjects and in the patients before and after the operation. The post-operative serum samples were collected simultaneously with the pleural fluid samples. The average serum HGF level in healthy volunteers was 0.36 ± 0.13 ng $ml^{-1}$ ($n$ = 50). The patients' serum HGF concentrations before and after operation were 0.61 ± 0.33 and 1.01 ± 0.72 ng $ml^{-1}$ respectively ($n$ = 9).

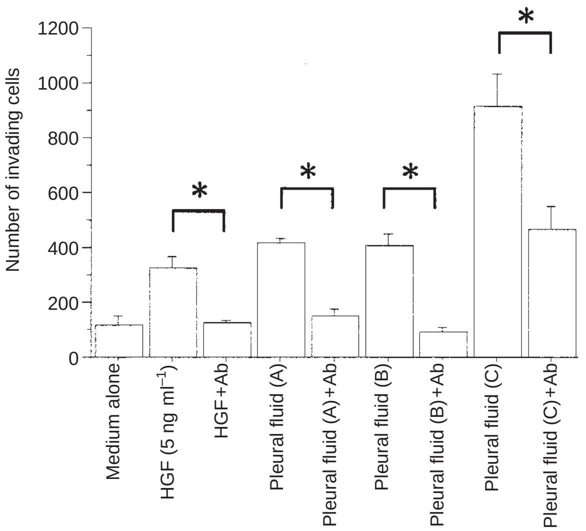

**Figure 6** Inhibition of invasion-stimulatory activity in the pleural fluid by HGF-neutralizing antibody. Antibody (2.5 μg ml$^{-1}$) was added to the lower compartment of the chamber with either the recombinant HGF (5 ng ml$^{-1}$) or diluted pleural fluids obtained from three different cases (one obtained on POD3 in case 4 (A), one on POD1 in case 3 (B), and one on POD3 after the third operation in case 6 (C)). **P* < 0.01

The HGF level in the pleural fluid was shown to be significantly higher than the serum HGF levels by the Kruskal–Wallis ANOVA test ($P < 0.0001$).

The HGF is known to stimulate invasion of carcinoma cells. To examine whether the pleural fluid can stimulate carcinoma cell invasion, serial dilutions of a pleural fluid sample obtained on POD 6 after the third operation in case 6 were tested for the effect on matrix invasion by a lung carcinoma cell line. The A549 line was positive for c-Met/HGF receptors as determined by reverse transcription polymerase chain reaction (RT-PCR) and Western blot analysis (data not shown). As shown in Figure 4, carcinoma cell invasion in the presence of medium alone and in the undiluted pleural fluid was 44.3 ± 5.2 and 306.3 ± 41.8 cells per 5 fields respectively. The difference between the two average values was significant ($P < 0.01$). Serial dilution (two-, three-, and fourfold) of the pleural fluid samples decreased the invasive response, suggesting the presence of invasion-stimulatory activity in the pleural fluid. Then we tested further seven pleural fluid samples from five cases for the effects on carcinoma cell invasion and compared them with the effects of culture medium and serum from four normal subjects (Figure 5). The matrix invasion by lung carcinoma cells was significantly ($P < 0.01$) increased in the presence of the pleural fluid (556.4 ± 236.9 cells per 5 fields) compared with that in the presence of culture medium (159.5 ± 76.9 cells per 5 fields) and normal sera (117.7 ± 94.4 cells per 5 fields).

To examine whether the HGF in the pleural fluid contributes to invasion-stimulatory activity, HGF-neutralizing antibody against HGF (2.5 μg ml$^{-1}$) was added to the lower compartment of the chamber with pleural fluid samples obtained from three cases (one obtained on POD 3 in case 4 [A], one on POD 1 in case 3 [B], and one on POD 3 of the third operation in case 6 [C]). Each sample was diluted, and the HGF concentration in the lower compartment was adjusted to 3.75, 4.75, or 5.50 ng ml$^{-1}$ in the pleural fluid A, B, or C respectively. The A549 lung carcinoma cells invaded the Matrigel in response to 5 ng ml$^{-1}$ of the recombinant human HGF. To confirm the activity of the HGF-neutralizing antibody, the antibody (2.5 μg ml$^{-1}$) was added in the lower compartment with 5 ng ml$^{-1}$ recombinant HGF. As shown in Figure 6, the antibody completely blocked the invasion stimulated by the HGF. The HGF-neutralizing antibody almost completely blocked the carcinoma cell invasion in response to the pleural fluids A and B, and significantly blocked the invasion in response to the pleural fluid C (Figure 6).

The relationship between HGF level and invasion-stimulatory activity (number of invading carcinoma cells) among the seven pleural fluids and four normal sera was analysed. These samples were the same as those used in the experiments shown in Figure 5. A positive correlation was found ($P = 0.0073$).

### DISCUSSION

We found that high concentrations of HGF are secreted in the pleural fluid after lung surgery, that pleural fluid can stimulate carcinoma cell invasion in vitro, and that HGF mediates such activity at least in part. Although such activity was found in vitro, these results suggest that the local HGF induced by lung surgery may contribute to invasive progression of residual carcinoma cells.

Kenworthy et al (1992) and Eagles et al (1996) have previously reported the presence of HGF in malignant pleural effusions. They showed the presence of HGF by both ELISA and biological assay for cell scattering activity, and suggested that HGF may contribute to metastatic spread in the pleura. We have shown the presence and activity of the HGF in pleural fluid after lung surgery. Stimulation of chemoinvasion by carcinoma cells is a unique characteristics of HGF (Giordano et al, 1993), and matrix invasion by cancer cells is an important step in metastatic behaviour. The median HGF levels in pleural effusion fluids from lung cancer patients demonstrated by Eagles et al (1996) was 0.49 ng ml$^{-1}$. On the other hand, the HGF levels in the pleural fluids after lung surgery in the current study ranged from 6.0 to 23.0 ng ml$^{-1}$ with an average of 10.1 ng ml$^{-1}$. The difference of the HGF concentration in the pleural fluid between these pre- and post-operative studies is remarkable. Together with the data of time course of HGF concentration in the pleural fluid after surgery, it is suggested that the HGF concentration in the pleural fluid increased following lung surgery. The level of HGF in pleural fluid after surgery was high, average being 10.1 ng ml$^{-1}$. A number of in vitro experiments have shown that concentrations of HGF above 1 ng ml$^{-1}$ stimulate motility and invasion of carcinoma cells expressing the HGF receptors (Uchiyama et al, 1996; Nakamura et al, 1997). The HGF level in pleural fluid after lung surgery cannot be negligible for it to have an effect on invasion of residual carcinoma cells.

The identification of the HGF in the post-operative pleural fluid prompted us to investigate the invasive response of lung carcinoma cells to the pleural fluid. As expected, the pleural fluid stimulated matrix invasion by the lung carcinoma cells in vitro. This was evidenced by the findings that the invasive response in the presence of the pleural fluid was reduced by serial dilution of the fluid, and that sera from normal subjects had significantly lower activity to stimulate carcinoma cell invasion. The in vitro invasion assay in this study has been used to examine the invasion of carcinoma cells across the reconstituted basement membrane in response to chemoattractants present in the pleural fluid. A number of cytokines, or growth factors, are thought to be present in the pleural fluid. Among them, the HGF is known to be a unique growth factor that can stimulate carcinoma cell invasion. In our

© 1999 Cancer Research Campaign experiments, HGF-neutralizing antibody inhibited carcinoma cell invasion in response to the pleural fluid. Positive correlation was also found between HGF level and invasion-stimulatory activity in the pleural fluid. Although other cytokines may also be responsible for the enhanced invasion of the carcinoma cells, the HGF is thought to be one of the key invasion-stimulatory factors in the microenvironments of lung surgery.

The actual mechanism of HGF secretion into the pleural fluid is uncertain. One of the possible sources of the HGF is lung fibroblasts (Weidner et al, 1990). It has been shown that the HGF is produced by fibroblasts stimulated with inflammatory cytokines (Matsumoto et al, 1992, 1996; Nakamura et al, 1997). Serum HGF levels are elevated in patients who underwent hepatectomy (Tomiya et al, 1992; Kimura et al, 1996), patients with systemic inflammatory response syndrome (Sakon et al, 1996) and patients with acute pancreatitis (Ueda et al, 1996). These reports suggest that secretion of HGF is closely related to the occurrence of inflammation. Cancer surgery, including organ resection or lymph node dissection, causes extensive damage to stromal tissue. A series of inflammatory reactions enabling tissue repair after lung surgery may play a role in the secretion of HGF. The other possible sources of the HGF in the lung are endothelial cells in lung parenchyma and alveolar macrophages (Yanagita et al, 1992). Since expression of HGF increases following lung injury and HGF enhances lung regeneration (Yanagita et al, 1993; Ohmichi et al, 1996), the increase of HGF in pleural fluid may possibly reflect physiological response to enhance the tissue repair process.

Post-operative cancer invasion and metastasis are critical clinical problems in the surgical treatment of cancer (Kodama et al, 1992; Nomoto et al, 1996). It has been shown in an animal model that the seeding of blood-borne metastases to the lung was significantly enhanced by surgical wounding of normal tissues (Bogden et al, 1997). Case 1 in our series had a sulcus tumour in the right apex of the lung. Pathological examination of the resected specimen showed that residual carcinoma cells were present in the surgical margin of the pleura. A follow-up computerized tomography examination showed the residual carcinoma invading the right brachial nerve plexus and thoracic vertebrae only 3 months after surgery. The expression of c-Met/HGF receptor mRNA and protein in the resected carcinoma tissue was identified by RT-PCR and Western blotting analysis. In this case, it is highly probable that the invasive growth of residual carcinoma cells was enhanced by HGF and invasion-stimulatory activity in the pleural fluid. Although further studies are needed to verify the actual roles of the HGF in clinical cancer invasion and metastasis, surgical oncologists should be well aware of the fact that lung surgery may provide a suitable microenvironment for invasion by residual carcinoma cells in some cases.

### ACKNOWLEDGEMENTS

We thank Ms. Sakamoto for expert assistance. This work was supported in part by a Grant in Aid for Scientific Research from the Ministry of Education, Science and Culture, Japan.

### REFERENCES

Bogden AE, Moreau JP and Eden PA (1997) Proliferative response of human and animal tumours to surgical wounding of normal tissues: onset, duration and inhibition. *Br J Cancer* **75**: 1021–1027

Eagles G, Warn A, Ball RY, Baillie-Johnson H, Arakaki N, Daikuhara Y and Warn RM (1996) Hepatocyte growth factor/scatter factor is present in most pleural effusion fluids from cancer patients. *Br J Cancer* **73**: 377–381

Giordano S, Zhen Z, Medico E, Gaudino G, Galimi F and Comoglio PM (1993) Transfer of motogenic and invasive response to scatter factor/hepatocyte growth factor by transfection of human *MET* protooncogene. *Proc Natl Acad Sci USA* **90**: 649–653

Jeffers M, Rong S and Woude GF (1996) Enhanced tumorigenicity and invasion-metastasis by hepatocyte growth factor/scatter factor-Met signalling in human cells concomitant with induction of the urokinase proteolysis network. *Mol Cell Biol* **16**: 1115–1125

Kenworthy P, Dowrick P, Baillie-Johnson H, McCann B, Tsubouchi H, Arakaki N, Daikuhara Y and Warn RM (1992) The presence of scatter factor in patients with metastatic spread to the pleura. *Br J Cancer* **66**: 243–247

Kimura F, Miyazaki M, Suwa T, Kakizaki S, Itoh H, Kaiho T, Ambiru S, Shimizu H and Togawa A (1996) Increased levels of human hepatocyte growth factor in serum and peritoneal fluid after partial hepatectomy. *Am J Gastroenterol* **91**: 116–121

Kodama M, Kodama T, Nishi Y and Totani R (1992) Does surgical stress cause tumor metastasis? *Anticancer Res* **12**: 1603–1616

Matsumoto K and Nakamura T (1997) Hepatocyte growth factor (HGF) as a tissue organizer for organogenesis and regeneration. *Biochem Biophys Res Commun* **239**: 639–644

Matsumoto K, Okazaki H and Nakamura T (1992) Up-regulation of hepatocyte growth factor gene expression by interleukin-1 in human skin fibroblasts. *Biochem Biophys Res Commun* **188**: 235–243

Matsumoto K, Date K, Shimura H and Nakamura T (1996) Acquisition of invasive phenotype in gallbladder cancer cells via mutual interaction of stromal fibroblasts and cancer cells as mediated by hepatocyte growth factor. *Jpn J Cancer Res*, **87**: 702–710

Miyazawa K, Tsubouchi H, Naka D, Takahashi K, Okigaki M, Arakaki N, Nakayama H, Hirono S, Sakiyama O, Gohda E, Daikuhara Y and Kitamura N (1989) Molecular cloning and sequence analysis of cDNA for human hepatocyte growth factor. *Biochem Biophys Res Commun*, **163**:967–973

Nakamura T, Nawa K and Ichihara A (1984) Partial purification and characterization of hepatocyte growth factor from serum of hepatectomized rats. *Biochem Biophys Res Commun* **122**: 1450–1459

Nakamura T, Nishizawa T, Hagiya M, Seki T, Shimonishi M, Sugimura A, Tashiro K and Shimizu S (1989) Molecular cloning and expression of human hepatocyte growth factor. *Nature* **342**: 440–443

Nakamura T, Matsumoto K, Kiritoshi A, Tano Y and Nakamura T (1997) Induction of hepatocyte growth factor in fibroblasts by tumor-derived factors affects invasive growth of tumor cells: in vitro analysis of tumor-stromal interactions. *Cancer Res* **57**: 3305–3313

Noji S, Tashiro K, Koyama E, Nohno T, Ohyama K, Taniguchi S and Nakamura T (1990) Expression of hepatocyte growth factor gene in endothelial and Kupffer cells of damaged rat livers, as revealed by in situ hybridization. *Biochem Biophys Res Commun* **173**: 42–47

Nomoto S, Nakao A, Kasai Y, Harada A, Nonami T and Takagi H (1996) Detection of *ras* gene mutations in perioperative peripheral blood with pancreatic adenocarcinoma. *Jpn J Cancer Res* **87**: 793–797

Ohmichi H, Matsumoto K and Nakamura T (1996) In vivo mitogenic action of hepatocyte growth factor on bronchial epithelial cells: pulmotrophic role of HGF in lung regeneration. *Am J Physiol* **270**: L1031–L1039

Sakon M, Kita Y, Yoshida T, Umeshita K, Gotoh M, Kanai T, Kawasaki T, Kambayashi J and Monden M (1996) Plasma hepatocyte growth factor levels are increased in systemic inflammatory response syndrome. *Surg Today* **26**: 236–241

Siegfried JM, Weissfeld LA, Singh-Kaw P, Weyant RJ, Testa JR and Landreneau RJ (1997) Association of immunoreactive hepatocyte growth factor with poor survival in resectable non-small cell lung cancer. *Cancer Res* **57**: 433–439

Sieuwerts AM, Klijn JG and Foekens JA (1997) Assessment of the invasive potential of human gynecological tumor cell lines with the in vitro Boyden chamber assay: influences of the ability of cells to migrate through the filter membrane. *Clin Exp Metastasis* **15**: 53–62

Sonnenberg E, Meyer D, Weidner KM and Birchmeier C (1993) Scatter factor/hepatocyte growth factor and its receptor, the c-met tyrosine kinase can mediate a signal exchange between mesenchymal and epithelia during mouse development. *J Cell Biol* **123**: 223–235

Tomiya T, Tani M, Yamada S, Hayashi S, Umeda N and Fujiwara K (1992) Serum hepatocyte growth factor levels in hepatectomized and nonhepatectomized surgical patients. *Gastroenterology* **103**: 1621–1624

Uchiyama A, Essner R, Doi F, Nguyen T, Ramming KP, Nakamura T, Morton DL and Hoon DSB (1996) Interleukin 4 inhibits hepatocyte growth factor-induced invasion and migration of colon carcinomas. *J Cell Biochem* **62**: 443–453

© 1999 Cancer Research Campaign 

Ueda T, Takeyama Y, Toyokawa A, Kishida S, Yamamoto M and Saitoh Y (1996) Significant elevation of serum human hepatocyte growth factor levels in patients with acute pancreatitis. *Pancreas* **12**: 76–83

Weidner KM, Behrens J, Vandekerckhove J and Birchmeier W (1990) Scatter factor: molecular characteristics and effect on the invasiveness of epithelial cells. *J Cell Biol* **111**: 2097–2108

Yanagita K, Nagaike M, Ishibashi H, Niho Y, Matsumoto K and Nakamura T (1992) Lung may have an endocrine function producing hepatocyte growth factor in response to injury of distant organs. *Biochem Biophys Res Commun* **182**: 801–809

Yanagita K, Matsumoto K, Sekiguchi K, Ishibashi H, Niho Y and Nakamura T (1993) Hepatocyte growth factor may act as a pulmotrophic factor on lung regeneration after acute lung injury. *J Biol Chem* **268**: 21212–21217

Zarnegar R and Michalopoulos GK (1995) The many faces of hepatocyte growth factor: from hepatopoiesis to hematopoiesis. *J Cell Biol* **129**: 1177–1180

 *© 1999 Cancer Research Campaign*
